# Biomarkers Related to Carotid Intima-Media Thickness and Plaques in Long-Term Survivors of Ischemic Stroke

**DOI:** 10.1007/s12975-015-0403-0

**Published:** 2015-05-08

**Authors:** Ulrike Waje-Andreassen, Halvor Naess, Lars Thomassen, Tove Helene Maroy, Kibret Yimer Mazengia, Geir Egil Eide, Christian Alexander Vedeler

**Affiliations:** Department of Neurology, Haukeland University Hospital, Jonas Lies vei 65, N-5021 Bergen, Norway; Department of Clinical Medicine, University of Bergen, Bergen, Norway; Centre for Clinical Research, Haukeland University Hospital, Bergen, Norway; Lifestyle Epidemiology Research Group, Department of Global Public Health and Primary Care, University of Bergen, Bergen, Norway

**Keywords:** Young ischemic stroke, Long-term follow-up, Carotid IMT, Fc gamma receptors, IL-10, HbA1c

## Abstract

Lifestyle risk factors, inflammation and genetics play a role in the development of atherosclerosis. We therefore studied Fc gamma receptor (FcγR) polymorphisms, interleukin (IL)-10 polymorphisms and other biomarkers related to carotid intima-media thickness (cIMT) in patients with ischemic stroke at a young age. Patients were evaluated 12 years after stroke occurrence. Patients (*n* = 232) 49 years of age or younger with an index stroke between 1988 and 1997 were retrospectively selected. Blood samples were taken at a first follow-up 6 years after the stroke. At a second follow-up, additional arterial events were registered for 140 patients, new blood samples were taken, and measurements of cIMT and blood pressure (BP) were performed. Unadjusted logistic regression analysis showed that cIMT ≥1 mm was associated with age, male gender, additional arterial events, BP, cholesterol, sedimentation rate, haemoglobin, triglycerides, creatinine, glycolysed haemoglobin (HbA1c) and FcγRIIIB-NaII/NaII. Adjusted backward stepwise logistic regression showed significance for age (odds ratio (OR) 1.13, 95 % confidence interval (CI) 1.04 to1.23, *p* = 0.003), male gender (OR 4.07, 95 % CI 1.15 to 14.5, *p* = 0.030), HbA1c (OR 6.65, 95 % CI 1.21 to 36.5, *p* = 0.029) and FcγRIIIB-NaII/NaII (OR 3.94, 95 % CI 1.08 to 14.3, *p* = 0.037). In this long-term follow-up study of patients with ischemic stroke at a young age, FcγRIIIB-NaII/NaII was identified as a possible contributing factor for cIMT ≥1 mm together with known risk factors, such as age, male gender, systolic BP, additional arterial events and HbA1c.

## Introduction

Atherosclerosis is an inflammatory disease with both lifestyle and genetic risk factors. Receptors for the Fc portion of immunoglobulin G (IgG), known as FcγRs, are the binding link between humoral and cellular immunologic reactions. Endothelial cells express FcγRs and pro-inflammatory mediators, such as immune complexes and C-reactive protein, that can activate FcγR-dependent pathways that lead to oxidative burst, degranulation, phagocytosis, cytokine production and antibody-dependent cell-mediated cytotoxicity (ADCC) [1]. The FcγRs are surface glycoproteins, encoded by eight genes located on chromosome 1q21–23. Based on structural homology and differences in affinity for IgG, this family is divided into three subfamilies: FcγRI (CD64), FcγRII (CD32) and FcγRIII (CD16). FcγR isoforms can be activating receptors (FcRI, FcγRIIA and FcγRIII) or inhibitory receptors (FcγRIIB) [1]. Two studies have demonstrated associations between certain FcγRIIA polymorphisms and coronary artery disease (CAD) and ischemic stroke [2, 3]. However, a study of patients with myocardial infarction and patients with CAD, documented by angiography, did not confirm this association [4]. Protective effects have been reported for the FcγRIIA polymorphism and peripheral atherosclerosis [5], and an inverse relation has been found for FcγRIIIA and CAD [6].

Interleukin (IL)-10 is another important immune regulator that may be important in the pathogenesis of atherosclerosis [7]. Low levels of expression of IL-10 are associated with atherosclerosis and cardiac and vascular dysfunction in mouse models [8, 9], and in humans, reduced levels of IL-10 are associated with carotid atherosclerosis and coronary disease [10, 11]. IL-10 production varies due to single-nucleotide polymorphisms (SNPs) in the promoter region of the IL-10 gene [12]. IL-10 SNPs have been identified at positions −1082 (A/G), −819 (C/T) and −592 (A/C) in Caucasians and form the haplotypes ATA, ACC and GCC in six possible combinations [13]. The genotype GCC/GCC is associated with high production of IL-10; GCC/ACC and GCC/ATA with medium production; and ATA/ATA, ATA/ACC and ACC/ACC with low production of IL-10 [14]. Although the IL-10 gene is also localised on chromosome 1, the polymorphisms of IL-10 and FcγR are not related. However, it has been shown that cross-linking of FcγRs on macrophages influence the IL-10 production [15].

We have previously reported data from 232 patients who had suffered acute arterial ischemic stroke at ages between 15 and 49 years and found a 10-fold higher mortality rate and a 5-fold increased vascular morbidity rate among long-term survivors compared with controls [16]. After a mean observation time of 12 years from the index stroke, individual maximum carotid intima-media thickness (cIMT) values were ≥1.0 mm in 76 % of 140 examined patients, based on 5944 wall-segmental measurements [17].

In the present study, we analysed these retrospectively selected young stroke patients with the following biomarkers: C-reactive protein (CRP), homocysteine, cholesterol and triglycerides, sedimentation rate (SR), haemoglobin (Hb), glycolysed haemoglobin (HbA1c), creatinine, leukocytes, thrombocytes, and FcγR polymorphisms, IL-10 polymorphisms and *Chlamydia pneumoniae* antibodies. For multivariate analysis, we related these parameters and other relevant clinical information, such as age, gender, blood pressure, number of smoking years and additional arterial events other than the index stroke to cIMT.

## Methods

### Patients and Study Design

**Fig. 1:**
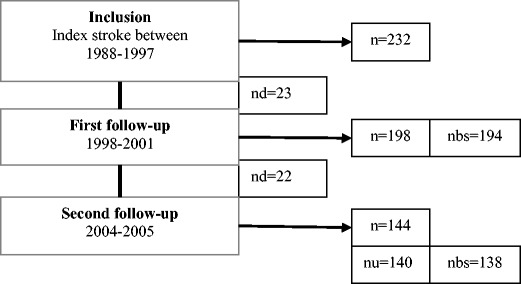
Flow chart of 232 retrospectively included ischemic stroke patients after the index stroke at age 17 to 49 years; *n* = number of patients who were identified and participated in follow-ups, *nd* = numbers of patients who died between inclusion and first follow-up and between first and second follow-up, *nbs* = number of collected blood samples. Four patients at first follow-up and two patients at second follow-up refused blood sampling, but both patients from second follow-up permitted the use of blood samples from other health controls, done within 1 month; *nu* = number of patients with carotid ultrasound measurements

We identified 232 patients of less than 50 years of age with first-ever ischemic stroke between 1988 and 1997 from five acute care hospitals in Hordaland County in western Norway [16]. The mean age (standard deviation, SD) of the subjects was 41.1 (±7.5) years at the time of the index stroke. Ischemic stroke was defined according to the Baltimore Washington Cooperative Young Stroke Study Criteria as neurological deficits lasting longer than 24 h or clinical transient ischemic attacks (TIAs) in which cerebral computed tomography (CT) or magnetic resonance imaging (MRI) showed acute arterial cerebral infarction related to clinical findings [18]. About two thirds of the patients had anterior circulation infarction, about one third had posterior circulation infarction, and despite more serious affection of patients with anterior circulation infarction, the short-term outcome at discharge was favourable with modified Rankin score ≤2 in 80 % [19]. From 1988 to 1997, patients were mainly treated by platelet inhibition or anticoagulation. Thrombolysis and treatment with statins were not usual at that time. Figure 1 shows the flow chart of the study. The first follow-up was done from 1998 to 2001 after a mean time of 5.7 years from the index stroke. Blood samples were taken from 198 participating patients and frozen at −80 °C. Four patients refused blood sampling. A second follow-up was done from 2004 to 2005 after a mean time of 11.9 years from the index stroke. Until the second follow-up, 45 patients had died, mainly from cardiovascular disease [20]. Of the 187 survivors, 144 (77 %) participated in clinical examinations, and 140 patients were examined with cIMT measurements by B-mode ultrasound [17]. Two patients refused blood sampling but permitted the use of samples taken for other control purposes within 1 month. The mean age at this follow-up was 52.9 years (SD ± 8.1, range 28–65 years).

### FcγR Polymorphisms

FcγR polymorphisms were analysed as previously described [6]. In brief, leukocytes were isolated from peripheral venous blood samples (EDTA blood). DNA was extracted with QIAampTM Blood Kit (Qiagen GmbH, Hilden, Germany). FcγR genotypes were determined by polymerase chain reaction (PCR), and all PCR products were analysed by electrophoresis [6].

### IL-10 Polymorphisms

DNA was extracted from whole-blood samples and examined for IL-10 polymorphisms at positions −1082 (G/A), −819 (T/C) and −592 (A/C) using PCR and gene sequencing as previously described [21]. The IL-10 haplotypes were determined and grouped into combinations with high IL-10 expression, medium IL-10 expression and low IL-10 expression.

### Carotid Artery Imaging

Right- and left-sided cIMT examination of near and far walls of the distal common carotid artery (CCA), bifurcation (BIF) and the proximal internal carotid artery (ICA) were performed at four predefined angles. CCA, BIF and ICA vessel segments were identified by using the tip of the flow divider as an internal landmark. Segments had a length of 10 mm, and the mean value was measured. A total of 5944 segmental mean IMT or plaque values were obtained. The maximum value of 48 possible individual cIMT measurements was used as outcome variable. cIMT results were used to divide subjects into two cIMT categories: <1.0 and ≥1.0 mm. Our 140 patients had a mean age of 53 years, the oldest patients were 65 years old at that final clinical follow-up, and total maximum IMT of 1.0 mm was chosen as measure of dichotomisation due to literature [22, 23]. Methods, results and patients’ characteristics were previously described in detail [17].

### Data Analysis

Biomarkers were compared between the groups with cIMT <1 mm and cIMT ≥1 mm. Age, additional arterial events, systolic and diastolic BP, number of smoking years, SR, HbA1c, creatinine, Hb, leukocytes, thrombocytes and triglycerides were used as results from the second follow-up. CRP, homocysteine, total cholesterol, LDL cholesterol and HDL cholesterol were used as results from the first and second follow-up. Categorical variables were analysed with 3×2 chi-square test and Fisher’s exact test, and Wilcoxon–Mann–Whitney test was used for continuous variables. Unadjusted logistic regression analysis of the dichotomised cIMT was used to evaluate the potential relationships with FcγR polymorphisms, IL-10 haplotypes with ATA/ATA as reference variable and with *C. pneumoniae* antibodies. *p* values were calculated from the likelihood ratio test. Multiple logistic regression analysis was done using the dichotomised cIMT results as outcome in a backward stepwise selection process retaining variables significant at the 5 % level in the final model. Multiple linear regression analysis was done using continuous maximum cIMT results as outcome in a backward stepwise selection process retaining variables significant at the 5 % level in the final model. The analysis was performed using the Statistical Package for Social Sciences (SPSS) 20. *p* values <0.05 were considered significant.

## Results

### Immunological Parameters and cIMT

Table 1 shows FcγRIIIB polymorphisms associated with cIMT ≥1 mm. Table 2 shows total cholesterol, LDL cholesterol and homocysteine from the first follow-up associated with cIMT ≥1 mm, and further association with Hb, HbA1c and creatinine from the second follow-up. Age, gender, systolic and diastolic BP and additional arterial events were also associated with cIMT ≥1 mm (Table 2). We found a significant association between the FcγRIIIB NAII/NAII polymorphism and cIMT ≥1 mm by using unadjusted logistic regression and adjusted backward stepwise logistic regression analysis (Table 3). However, linear regression analysis showed non-significant results for the FcγRIIIB NAII/NAII polymorphism relation with continuous cIMT. There was also a significant association between the IL-10 GCC/ACC polymorphisms and cIMT ≥1 mm, but this was not correlated with other polymorphisms associated with the degree of IL-10 production (Table 1). Therefore, we did not follow up the IL-10 results by performing final backward stepwise logistic and linear regression analysis. *C. pneumoniae* antibodies, sedimentation rate and C-reactive protein were not associated with cIMT ≥1 mm.

**Table 1:** Fc gamma receptor (FcγR) polymorphisms and interleukin (IL)-10 polymorphisms related to carotid intima-media thickness (cIMT) with ≥1 mm as outcome variable

Immunology				
Variables	Number %	cIMT <1 mm *n* = 34	cIMT ≥1 mm *n* = 104	LR test *p* value
Fc gamma receptor (FcγR) polymorphisms				
FcγRIIA	*n* = 138			0.313
H/H	15.9	5	17	
H/R	42.0	18	40	
R/R	42.0	11	47	
FcγRIIIB	*n* = 138			0.036
NaI/NaI	12.0	6	10	
NaI/NaII	43.5	19	41	
NaII/NaII	44.9	9	53*	
FcγRIIIA	*n* = 137			0.362
*V/V*	12.4	2	15	
V/F	40.9	15	41	
F/F	46.7	17	47	
Interleukin (IL)-10 polymorphisms				
	*n* = 138			0.102
ATA/ATA	7.2	4	6	
ATA/ACC	9.4	5	8	
ACC/ACC	7.2	1	9	
GCC/ACC	26.8	4	33*	
GCC/ATA	23.2	10	22	
GCC/GCC	26.1	10	26	
IL-10 producing genotypes [14]				
Low IL-10 production: ATA/ATA, ATA/ACC and ACC/ACC		10	23	0.353
Medium IL-10 production: GCC/ACC and GCC/ATA		14	55	0.238
High IL-10 production: GCC/GCC		10	26	0.655

*n* number, *LR* likelihood ratio test from logistic regression

*Significant *p* value, *p* ≤ 0.05

**Table 2:** Other clinical chemistry and clinical biomarkers related to carotid intima-media thickness (cIMT) ≥1 mm as outcome variable

	*n* of samples	cIMT <1 mm mean value (±SD)	cIMT ≥1 mm mean value (±SD)	*p* value
Data from first follow-up, 6 years after the index stroke				
*Chlamydia pneumoniae* antibodies	140	3	8	0.728
Homocysteine (μM)	139	12.8 (16.7)	11.4* (7.7)	0.011
CRP (mg/L)	134	2.1 (3.1)	2.5 (6.5)	0.344
Total cholesterol (mM)	139	5.5 (1.1)	6.1*(1.1)	0.007
LDL cholesterol (mM)	139	3.48 (0.9)	3.90* (1.0)	0.030
HDL cholesterol (mM)	139	1.33 (0.4)	1.29 (0.4)	0.778
Data from second follow-up, 12 years after the index stroke				
CRP (mg/L)	139	2.6 (3.0)	3.7 (5.7)	0.344
Sedimentation rate (mm)	135	8.8 (6.3)	12.9 (10.1)	0.055
Homocysteine (μM)	132	11.8 (10.4)	12.4 (7.5)	0.098
Total cholesterol (mM)	139	5.37 (1.0)	5.47 (1.2)	0.590
LDL-cholesterol (mM)	138	3.41 (0.9)	3.54 (1.1)	0.543
HDL-cholesterol (mM)	140	1.64 (0.5)	1.51 (0.4)	0.331
Triglycerides (mM)	137	1.43 (0.6)	1.75 (0.8)	0.065
Haemoglobin (g/dL)	139	14.0 (1.1)	14.6* (1.3)	0.024
Glycolysed haemoglobin (%)	135	5.4 (0.7)	5.8* (0.9)	<0.001
Creatinine (μM)	138	64.3 (13.6)	83.1* (70.7)	<0.001
Leukocytes (10^9^/L)	140	7.3 (2.2)	7.2 (1.9)	0.948
Thrombocytes (10^9^/L)	138	268 (55.4)	262 (74.6)	0.221
Other variables at second follow-up				
Age (years)	140	45.6 (9.9)	55.2* (5.8)	<0.001
Gender			*	0.001
Males (*N*)		10	68	
Females (*N*)		24	38	
Blood pressure (mmHg)	137			
Systolic		131 (18.4)	143* (16.8)	0.001
Diastolic		84 (11.1)	90* (9.9)	0.002
*n* of smoking years	53 active and 54 ex-smokers	22.3 (10.0)	26.9 (10.2)	0.078
AAE		6	46*	0.008

3×2 chi-square test and Fisher’s exact test for categorical variables and Wilcoxon–Mann–Whitney test for continuous variables were used for comparison of data. Mean value (±SD)

*n* number, *SD* standard deviation, *μM* micromole, *CRP* C-reactive protein, *LDL* low density lipoprotein, *HDL* high density lipoprotein, *AAE* additional arterial event other than the index stroke (recurrent ischemic stroke, angina pectoris, myocardial infarction, peripheral artery disease)

**Table 3:** Significant results of unadjusted logistic regression and adjusted backward stepwise logistic regression analysis, related to maximum carotid intima-media thickness, categorised as <1 and ≥1 mm in 140 patients, measured 12 years after acute ischemic stroke

	Unadjusted logistic regression			Adjusted logistic regression		
Variables	OR	(95 % CI)	*p* values	OR	(95 % CI)	*p* values
FcγRIIIB NaI/NaI	1.00					
FcγRIIIB NaII/NaII	2.78	1.18, 6.52	0.019	3.94	1.08, 14.3	0.037
Cholesterol	1.62	1.12, 2.34	0.010	–	–	–
SR	1.06	1.01, 1.12	0.031	–	–	–
Hb	1.41	1.02, 1.95	0.036	–	–	–
HbA1c	3.35	1.27, 8.86	0.015	6.65	1.21, 36.5	0.029
Triglycerides	1.75	1.00, 3.07	0.049	–	–	–
Creatinine	1.05	1.02, 1.08	0.001	–	–	–
Age	1.17	1.10, 1.25	<0.001	1.13	1.04, 1.23	0.003
Male gender	4.30	1.86, 9.93	0.001	4.07	1.15, 14.5	0.030
Systolic BP	1.05	1.02, 1.08	0.002	–	–	–
Diastolic BP	1.06	1.01, 1.10	0.008	–	–	–
AAE	3.64	1.39, 9.53	0.009	–	–	–

Unadjusted univariate logistic regression analysis was performed for all blood samples from the first and second follow-up: FcγR polymorphisms, IL-10 polymorphisms, *Chlamydia pneumoniae* antibodies, homocysteine, C-reactive protein, total cholesterol, LDL cholesterol, HDL cholesterol, SR, triglycerides, Hb, HbA1c, creatinine, leukocytes and thrombocytes and for clinical variables, such as age, gender, systolic BP, diastolic BP and AAE. Only significant found variables are listed and used in the adjusted backward stepwise logistic regression analysis

*OR* odds ratio, *CI* confidence interval, *FcγR* Fc gamma receptor polymorphism, *SR* sedimentation rate, *Hb* haemoglobin, *HbA1c* glycolysed haemoglobin, *BP* blood pressure, *AAE* additional arterial events (recurrent stroke and/or angina pectoris and/or myocardial infarction and/or peripheral artery disease) other than the index stroke

### Non-immunological Parameters and cIMT

cIMT ≥1 mm was associated with total cholesterol, LDL cholesterol and homocysteine at the first, but not at the second, follow-up (Table 2). Haemoglobin, HbA1c and creatinine were also associated with cIMT ≥1 mm as were age, male gender, systolic and diastolic BP and additional arterial events other than the index stroke. HbA1c was associated with cIMT ≥1 mm in addition to age and male gender after unadjusted logistic regression and results after adjusted backward stepwise logistic regression analysis (Table 3). Final linear regression analysis for continuous cIMT showed associations with age, additional arterial events, systolic BP and HbA1c (Table 4). Male gender was no longer associated with cIMT in that analysis.

**Table 4:** Final results of linear regression analysis, related to maximum carotid intima-media thickness (cIMT), measured in 140 patients 12 years after acute ischemic stroke

	b	Standard error (SE)	*p* values
Age	0.25	0.007	0.002
Systolic BP	0.18	0.003	0.029
AAE	0.20	0.111	0.019
HbA1c	0.18	0.061	0.028

Linear regression analysis was performed with Fc gamma receptor IIIB NaII/NaII polymorphism, total cholesterol, sedimentation rate, haemoglobin, HbA1c, triglycerides, creatinine, age, gender, systolic BP and AAE. Gender was non-significant in the final results (*b* = 0.13, SE = 0.1, *p* = 0.80)

*b* estimated regression coefficient, *BP* blood pressure, *AAE* additional arterial event other than the index stroke, such as recurrent ischemic stroke, angina pectoris, myocardial infarction and peripheral artery disease, *HbA1c* glycolysed haemoglobin

## Discussion

This is the first clinical study of long-term survivors of ischemic stroke at a young age to evaluate follow-up blood samples and carotid IMT measurements. The mean observation time was nearly 12 years, and the cIMT measurements also included plaques, as we measured at standardised sites in order to perform a staging of the carotid artery wall disease.

We have studied immunological and non-immunological risk factors for cIMT as parameters for carotid atherosclerosis. Our results showed no association with *C. pneumoniae* antibodies and cIMT. The association between *C. pneumoniae* antibodies and cardiovascular disease is unclear [24]. Furthermore, we did not find any association with cIMT and sedimentation rate or C-reactive protein. However, these parameters are crude markers for the evaluation of specific immune responses in the vascular wall.

We also studied the FcγR and IL10 genotypes as they have been linked to atherosclerosis. The frequencies of the FcγR and IL-10 genotypes among our patients were comparable to 272 healthy controls previously reported from western Norway [25, 26].

We found a significant association between FcγRIIIB NaII/NaII and cIMT ≥1 mm. However, linear regression analysis showed non-significant results for continuous maximum cIMT values. Others have reported that soluble FcγRIIIA is associated with maximum cIMT [27], that the FcγRIIIA VV-genotype is inversely related to the extent of coronary artery disease [6] and that the FcγRIIA R allele is associated with impaired endothelial vasodilation in patients with hypercholesterolemia [28].

The present study is the first to show a possible association with the low-affinity FcγRIIIB receptor and atherosclerosis as measured by cIMT ≥1 mm. FcγRIIIB, as well as FcγRIIA and FcγRIIIA, activates immune functions such as phagocytosis, ADCC and release of inflammatory mediators and superoxide radicals [29]. FcγRIIIB is only present on granulocytes, and the FcγRIIIB-NA2 allotype shows lower levels of phagocytosis of IgG1 and IgG3 opsonised particles than the NA1 allotype [30]. Our results therefore indicate that reductions in effector functions such as phagocytosis and ADCC may contribute to the development of atherosclerosis. Such findings are supported by knockout studies of the inhibitory FcγRIIB which shows an increased plaque formation [31]. In addition to myeloid cells, FcγRIIB is expressed on B cells where it suppresses B cell signaling and regulates IgG production [32]. Defects of FcγRIIB on B cell will therefore increase the IgG production, which was showed in this mouse model [31]. Our results indicate that similar mechanisms may take place in humans, as the NA2 allotype is associated with reduced phagocytosis of IgG1 and IgG3 [30].

Stimulation of activating FcγRs on macrophages has been found to induce an anti-inflammatory immune response with increased IL-10 production [15]. It has also been proposed that elevated IgG with IL-10 acting downstream is driving the plaque formation [31]. Others have reported that reduced levels of IL-10 and low-producing IL-10 genotypes are associated with increased cIMT [10, 33] and that low-producing IL-10 genotypes are associated with coronary disease [11] and ischemic stroke [34].

We have also studied the polymorphisms of IL-10 since 50–70 % of the variance in IL-10 levels is genetically determined [35]. However, we found no association between high-, medium- or low-producing IL-10 genotypes and cIMT ≥1 mm. Unfortunately, we had no access to serum IL-10 levels in our patients. Although we could not find any genetic risk factor with IL-10 and cIMT, this does not exclude the importance of this anti-inflammatory cytokine in the pathogenesis of atherosclerosis. The major roles of IL-10 in terms of atherosclerosis include inhibition of macrophage activation, as well as inhibition of matrix metalloproteinase, pro-inflammatory cytokines and cyclooxygenase-2 expression in lipid-loaded and activated macrophage foam cells. Furthermore, another important role of IL-10 in atherosclerosis is its ability to alter lipid metabolism in macrophages [36]. Of the non-immunological parameters, we found that cholesterol, triglycerides, blood pressure and cardiovascular events were risk factors in the unadjusted logistic regression analysis but not in the adjusted logistic regression analysis. However, these are known risk factors for subclinical carotid atherosclerosis [37, 38] At the first follow-up, statins were only used by 30 (15.5 %) of 194 patients with registered medication, and at the second follow-up, statins were used by 55 (38.2 %) of 144 patients [16]. The increasing use of statins probably explains why total cholesterol/homocysteine/and LDL-cholesterol levels were associated with cIMT at the first follow-up but not at the second follow-up.

We found that HbA1c, SR, haemoglobin and creatinine were risk factors in the unadjusted logistic regression analysis, but only HbA1c showed significance in the final adjusted logistic and linear regression analysis. Elevated blood glucose is a known risk factor for endothelial dysfunction and early atherosclerosis [39], and the high percentage of carotid artery atherosclerosis observed in our patients is in agreement with this. Furthermore, we found that increased age and male gender are correlated with increased cIMT as has been shown in large population studies [37, 40]. Surprisingly, male gender disappeared as significant result in the linear regression analysis, and other risk factors for increased cIMT and atherosclerosis, such as systolic BP and additional arterial events, became significant. Probably, the number of patients played a role for the varying results by the different methods of regression analysis.

The strength of our study is that we have a very well characterised patient population and clinically related immunological and non-immunological parameters were available for analysis. The frequencies of FcγR and IL-10 polymorphisms in our patients are similar to those observed in population studies performed from the same area [25, 26]. We have measurements of the various blood samples both at first and at second follow-up. In addition, we did meticulous studies to measure cIMT and plaques.

The weakness of our study is that patients were included retrospectively and selection was affected by death and a 23 % dropout rate of long-term survivors. The 23 % dropout rate at our last clinical follow-up, without any missing data concerning the dead-alive status, is, however, comparable to that of other clinical studies with follow-ups after 10 years [41].

Differences occurred after logistic and linear regression analysis concerning significancies related to cIMT in this study. However, age and HbA1c were in both analyses related to cIMT, and these parameters are well-known risk factors for increased cIMT and atherosclerosis. Male gender and increased BP are also well-known risk factors for atherosclerosis and additional arterial events, even if the results by the two types of regression analysis were slightly different. A new finding in this study is that FcγRIIIB-NAII/NAII appeared as a significant factor for increased cIMT ≥1 mm. However, further prospective studies with higher sample sizes are necessary to confirm these results.

In conclusion, we found that in patients with ischemic stroke at a young age, FcγRIIIB-NAII/NAII may be a contributing factor for increased cIMT together with known risk factors, such as age, male gender, systolic BP and increased HbA1c.
